# Leaving no one behind: a methodology for setting health inequality reduction targets for Sustainable Development Goal 3^*^

**DOI:** 10.26633/RPSP.2021.63

**Published:** 2021-04-28

**Authors:** Antonio Sanhueza, Isabel Espinosa, Oscar J. Mújica, Jarbas Barbosa da Silva Jr.

**Affiliations:** 1 Pan American Health Organization Washington, D.C. United States of America Pan American Health Organization, Washington, D.C., United States of America.

**Keywords:** Sustainable development, health equity, health status indicators, maternal mortality, Guatemala, Desarrollo sostenible, equidad en salud, indicadores de salud, maternal mortality, Guatemala, Desenvolvimento sustentável, equidade em saúde, indicadores básicos de saúde, mortalidade materna, Guatemala

## Abstract

**Objectives.:**

To present a methodology for the simultaneous setting of quantitative targets that reflect both an improvement in the national average of an indicator for Sustainable Development Goal 3 (SDG3), as well as a reduction in its geographic inequality.

**Methods.:**

A five-step algorithm was developed: (a) calculate the national average annual percent change (AAPC) for an SDG3 indicator; (b) normatively define geographic strata from the subnational distribution of the indicator in a baseline year; (c) apply a proportional progressivity criterion to the AAPC to project the stratum-specific indicator value for the target year; (d) set the national target as the weighted average of the indicator in the subnational territorial units for the target year; and (e) set the inequality reduction targets by calculating the absolute and relative gaps between the bottom and top strata for the target year.

**Results.:**

The algorithm was applied to SDG indicator 3.1.1 (maternal mortality ratio, MMR), disaggregated by Guatemala’s 22 departments at the baseline year 2014 (MMR = 113 per 100,000 live births). By sustaining the AAPC rate attained from 2009 to 2014 (-4.3%) and focalizing its actions with territorial progressivity, by 2030 the country could reduce its MMR to 53 per 100,000 and its absolute and relative inequality gaps by 72% and 48%, respectively.

**Conclusions.:**

The proposed methodology allows for simultaneously setting targets for overall progress and inequality reduction in health, making explicit the primacy of the equity principle contained in the SDG commitment to leave no one behind, whose urgency takes on renewed relevance in the current pandemic scenario.

## RESUMEN

**Objetivos.:**

Presentar una metodología para la formulación simultánea de metas cuantitativas que reflejen tanto la mejoría del promedio nacional de un indicador del tercer Objetivo de Desarrollo Sostenible (ODS3) como la reducción de su desigualdad geográfica.

**Métodos.:**

Se definió un algoritmo en cinco pasos: a) cálculo del cambio porcentual anual promedio (CPAP) nacional para un indicador del ODS3; b) definición normativa de estratos geográficos a partir de la distribución subnacional del indicador en un año base; c) aplicación de un criterio de progresividad proporcional del CPAP para proyectar el indicador estrato-específico al año meta; d) establecimiento de la meta nacional como el promedio ponderado del indicador en las unidades territoriales subnacionales al año meta; y e) formulación de metas de reducción de desigualdad mediante el cálculo de las brechas absoluta y relativa entre los estratos extremos al año meta.

**Resultados.:**

Se aplicó el algoritmo al indicador ODS 3.1.1 (razón de mortalidad materna, RMM), desagregado por los 22 departamentos de Guatemala para el año base 2014 (RMM = 113 por 100 000 nacidos vivos). Sosteniendo la intensidad promedio de CPAP observada entre 2009 y 2014 (-4,3%) y focalizando sus acciones con progresividad territorial, el país reduciría al 2030 su RMM a 53 por 100 000 nacidos vivos y sus brechas absoluta y relativa en 72% y 48%, respectivamente.

**Conclusiones.:**

La metodología propuesta permite formular simultáneamente metas de reducción de las desigualdades geográficas en salud y hacer explícita la primacía del principio de equidad expresado en el compromiso de *no dejar a nadie atrás *que identifica a los ODS, cuya urgencia cobra renovada relevancia en el escenario pospandémico actual.

## RESUMO

**Objetivos.:**

Apresentar uma metodologia para a formulação simultânea de metas quantitativas que reflitam tanto a melhoria da média nacional de um indicador do terceiro Objetivo de Desenvolvimento Sustentável (ODS3) quanto a redução das desigualdades geográfica nesse indicador.

**Métodos.:**

Estabelecemos um algoritmo em cinco etapas: (a) cálculo da variação percentual anual média (VPAM) em um país para um indicador do ODS3, (b) definição normativa de estratos geográficos a partir da distribuição subnacional do indicador em um ano base, (c) aplicação de um critério de progressividade proporcional da VPAM para projetar o indicador específico do estrato para o ano base, (d) estabelecimento da meta nacional como a média ponderada do indicador nas unidades territoriais subnacionais para o ano alvo e (e) estabelecimento de metas para a redução das desigualdades calculando a disparidade absoluta e relativa entre os estratos extremos para o ano alvo.

**Resultados.:**

Aplicamos o algoritmo ao indicador ODS 3.1.1 (razão de mortalidade materna, RMM), desagregado pelos 22 departamentos da Guatemala para o ano base de 2014 (RMM = 113 por 100.000 nascidos vivos). Se mantiver a intensidade média da VPAM observada entre 2009 e 2014 (-4,3%) e concentrar as suas ações com progressividade territorial, o país reduzirá, até 2030, a sua RMM para 53 por 100.000 e sua disparidade absoluta e relativa em 72% e 48%, respectivamente.

**Conclusões.:**

A metodologia proposta permite formular simultaneamente metas para a redução das desigualdades geográficas em saúde e explicitar a primazia do princípio da equidade expresso no compromisso de *não deixar ninguém para trás *consagrado nos ODS, cuja urgência assume uma relevância renovada no atual cenário pós-pandêmico.

Since September 2015, the world has a new global agenda for transforming people’s lives and their natural environment: the 2030 Agenda for Sustainable Development, which sets out a plan of action with 17 goals for global prosperity and peace (1). These goals, known as the Sustainable Development Goals (SDGs), are considered indivisible and are more comprehensive and ambitious than their predecessors, the Millennium Development Goals (MDGs). For instance, unlike the latter, the SDGs go beyond traditional poverty-related issues and include objectives related to peace, human rights, and good governance, as tracers of progress. The SDGs are organized in a common framework of 169 global targets and 244 global indicators (2). The third of these goals (SDG3) is to “ensure healthy lives and promote well-being for all at all ages,” the achievement of which means making good on the pledge to leave no one behind (1).

In contrast with this tacit recognition of universality and social equity as central elements in building opportunities for sustainable development, the SDG targets for 2030 have been formulated in a way similar to the MDG targets, as they consider only explicit changes in national and global indicator averages, without taking into account distributive inequality within countries. For example, the first SDG3 target (target 3.1) proposes: “By 2030, reduce the global maternal mortality rate to less than 70 per 100,000 live births” (2). Such a formulation, based on an average global value, is insufficient to track the trend of inequality (in this case, in the risk of maternal death), inform pro-equity public policies, and ensure accountability for the commitment to “leave no one behind” (3, 4).

In the countries of the Americas, a region of profound inequalities, the scale and trend of social inequalities in health—especially in the area of maternal and child health—have been documented for some years with growing interest, from both administrative and survey data (5–12). The analyses conducted have made it possible, on the one hand, to strengthen institutional capacities for the measurement and monitoring of social inequalities in health (13) and, on the other hand, to identify specific areas and population groups in situations of particular vulnerability, where health inequalities are disproportionately concentrated. These are the populations at risk of “being left behind.”

This article presents a methodology for simultaneously setting quantitative targets that reflect both improvement in the national average value for an SDG3 health indicator (average target) and the reduction of geographic inequality (distributional target).

## MATERIALS AND METHODS

### Data

The methodology requires the availability of SDG3 health indicator data geographically disaggregated at the subnational level (e.g., by state, department, province, canton, municipality, district, neighborhood or quarter) for a given period. These data may come from household surveys or administrative records. The level of geographic disaggregation (i.e., by territorial units) will depend on the availability and temporal consistency of the data.

### Methodology

The methodology consists of a five-step algorithm, called the target-setting algorithm for SDG3 (TSA_SDG3), which is explained below (Table 1).

#### Setting the average target

If the value of a health indicator (HI) at two defined times, *t_0_* and *t_1_*, is known, the average annual percent change (AAPC) in the indicator can be calculated using the following equation:

[1] $$AAPC=\frac{ln\left(HI_{t_{1}}\right)-ln\left(HI_{t_{0}}\right)}{\left(t_{1}-t_{0}\right)}\times 100$$

The value obtained from equation [1] reflects the rate of change in a health indicator over time. If the health indicator has negative polarity (i.e., when a lower value of the health indicator over time indicates a more favorable situation—e.g., mortality rate), the AAPC will show the average annual percentage *decrease*. If the health indicator has positive polarity (i.e., when a higher value of the health indicator over time indicates a more favorable situation—e.g., health care coverage), the AAPC will show the average annual percentage *increase*.

**TABLE 1.** Target-setting algorithm for Sustainable Development Goal 3 (TSA_SDG3)

Step 1: Calculate the national average annual percentage change (AAPC) of an SDG3 indicator from a known time series, with a baseline value and an annual reference value.^a^
Step 2: Define the geographic strata; to that end: ✓ Rank the geographic values of the health indicator at the baseline time by polarity (from highest to lowest if the indicator has negative polarity; from lowest to highest if it has positive polarity). ✓ Identify the cut points that define geographic strata, either by pre-established categories (e.g., above and below an established national reference value) or by groups according to quantiles (quintiles, quartiles, or terciles). ✓ Calculate the weighted average of the health indicator for each stratum thus defined.
Step 3: Apply the proportional progressivity criterion to the AAPC for each stratum defined; to that end: ✓ If the health indicator has negative polarity, assign an AAPC that is proportionally higher the higher the health indicator for the stratum, according to the proportionality factor. ✓ If the health indicator has positive polarity, assign an AAPC that is proportionally higher the lower the health indicator for the stratum, according to the proportionality factor. ✓ In any case, ensure that the arithmetic average of the AAPCs for all strata is equal to the national AAPC at the baseline time used in step 1.
Step 4: Set average targets at the subnational and national levels; to that end: ✓ Calculate the value of the health indicator for each territorial unit at a future time (subnational average target).^b^ ✓ Calculate the weighted average of the health indicator values for all territorial units at a future time (national average target). ✓ These results represent the targets at the subnational and national levels for the SDG3 indicator in absolute terms. The targets in relative terms are determined by applying equation [3].^c^
Step 5: Establish targets for reducing geographic inequality gaps; to that end: ✓ Calculate the absolute and relative inequality gaps (AG and RG) at baseline and future times.^d^ ✓ Calculate the percentage changes in AG and RG during the period.^c^ ✓ These results represent the targets for reducing geographic inequality gaps in the SDG3 indicator (distributional targets) in absolute and relative terms, respectively.

***Source:*** Prepared by the authors.

a See equation [1] in the text.

b See equation [2] in the text.

c See equation [3] in the text.

d See equations [4] and [5] in the text.

Assuming that the value of the AAPC and the value of the HI at a baseline time, *t_b_*, in each subnational geographic unit is known, a subnational target (i.e., for each geographic unit) for the HI at a future time, *t_f_*, can be established as follows (where *exp *is the mathematical constant of the exponential function):

[2] $${\mathrm{HI}}_{t_{f}}={\mathrm{HI}}_{t_{b}}\times \exp\left[\left(\frac{\mathrm{AAPC}}{100}\right)\times \left(t_{f}-t_{b}\right)\right]$$

The national average target is defined as the weighted average of the health indicator values in all territorial units at the future time *t_f_*. This weighted average represents a national target expressed in absolute terms, i.e., it is expressed in the same unit of measure as the health indicator. To express this target in relative terms—as a percentage of the value at the baseline time—the following equation is used to determine the percent change (PC) in the indicator between baseline time, *t_b_*, and future time, *t_f_*

[3] $$\mathrm{PC}=\frac{{\mathrm{HI}}_{t_{f}}-{\mathrm{HI}}_{t_{b}}}{{\mathrm{HI}}_{t_{b}}}\times 100$$

#### Setting the distributional target

Targets for reducing geographic inequality in health are expressed quantitatively by two standard metrics: the absolute inequality gap and the relative inequality gap (AG and RG, respectively), which are obtained from the defined subnational distribution of the health indicator for the target year, abridging it into geographic strata by applying a stratum-specific AAPC based on a proportional progressivity criterion.

AG and RG are simple summary measures of health inequality and represent the arithmetic difference and the arithmetic ratio, respectively, between the health indicator values for the bottom and top geographic strata (3, 13). If there are, for example, four geographic strata ordered according to a health indicator, the size of the geographic inequality gap is calculated as follows:

[4] $$\mathrm{AG}={\mathrm{HI}}_{q1}-{\mathrm{HI}}_{q4}$$

[5] $$\mathrm{RG}=\frac{{\mathrm{HI}}_{q1}}{{\mathrm{HI}}_{q4}}$$

where, *q_1_* is the first stratum (the stratum with the worst HI values) and *q_4_* is the fourth stratum (the stratum with best HI values).

The AG is expressed in the same unit of measure as the health indicator; AG = 0 denotes no inequality. The RG is expressed without units of measure, as its value represents the number of times the numerator is contained in the denominator; RG = 1 denotes no inequality (3, 13).

Once the subnational distribution has been ranked according to the status of the health indicator (by heuristic rule, from worst to best), the TSA_SDG3 proposes to abridge the distribution into geographic strata (depending on the number of territorial units at the subnational level, it is recommended to use two, three, four or, at most, five strata); apply a stratum-specific AAPC rate according to a proportional progressivity criterion; project their values for 2030 according to these AAPC rates; and calculate the absolute and relative inequality gaps for 2030. Strata are defined in accordance with normative criteria based on the observed national AAPC for the SDG3 health indicator.

The proportional progressivity criterion reflects the practical application of two related basic principles: the law of diminishing returns or marginal utility (the future improvement of a health indicator will be smaller the better its current status) (14) and the vertical equity principle (the contribution to the improvement of a health indicator should be greater the larger the margin for improvement–greater need) (15). From a normative standpoint, in order to operationalize the proportional progressivity criterion, the progressivity factor is set at 50%. If there are two strata (*q_1_* and *q_2_*), *q_1_* is assigned an AAPC that is 50% higher than the national AAPC and *q_2_* is assigned an AAPC that is 50% lower than the national AAPC. If there are three strata (*q_1_*, *q_2_*, and *q_3_*), the AAPC for *q_1_* is 50% higher than the national AAPC, the AAPC for *q_2_* is equal to the national AAPC, and the AAPC for *q_3_* is 50% lower than the national AAPC. If there are four strata, the stratum with the worst health situation, *q_1_*, is assigned an AAPC that is 50% higher than the national AAPC and subsequent strata are assigned AAPCs that are +25%, -25%, and 50% of the national AAPC. If there are five strata (*q_1_*, *q_2_*, *q_3_*, *q_4_*, and *q_5_*), the AAPC for *q_1_* is 50% higher than the national AAPC, the AAPC for *q_2_* is 25% higher than the national AAPC, the AAPC for *q_3_* is equal to the national AAPC, the AAPC for *q_4_* is 25% lower than the national AAPC, and the AAPC for *q_5_* is 50% lower than the national AAPC. In all cases, the average of all the specific AAPC values thus defined for each stratum is equal to the national AAPC.

## RESULTS

The proposed methodology was applied to subnational administrative data on the maternal mortality ratio (MMR) for Guatemala’s 22 departments in 2009 and 2014 (16). Nationally, the MMR was 140 per 100,000 live births (LB) in 2009 and 113 per 100,000 LB in 2014. When equation [1] is applied, the resulting national AAPC for the period is:

[6] $$\mathrm{AAPC}=\frac{\ln\left(113\right)-\ln\left(140\right)}{2014-2009}\times 100=-4.3\%$$

Based on Guatemala’s departmental MMR values in 2014 ranked from highest to lowest (Table 2), four geographic strata were defined according to two criteria: a national reference value of the MMR in 2030 calculated on the basis of the national AAPC (–4.3%) and the baseline MMR derived from equation [2] (57 per 100,000 LB) and a maximum threshold, equivalent to twice the global SDG3 target for this indicator (70 x 2 = 140 per 100,000 LB). The four strata group together departments with MMRs above the maximum threshold (stratum 1); under the threshold, but more than twice the national reference value (stratum 2); under twice the national reference value, but still above it (stratum 3); and under the national reference value (stratum 4).

The four strata thus defined were assigned an AAPC value reflecting proportional progressivity, based on the baseline national AAPC (–4.3%) and a preset progressivity factor of 50%. With these stratum-specific AAPC values, the MMR value for 2030 was projected for each department in the country—i.e., the average target for the department (Table 2). From this new departmental distribution of the MMR for 2030, the national MMR value was calculated, expressed as an average weighted by the number of live births in each department (projected live births for 2030); this weighted average represents the national average target for 2030 in absolute terms, which in this case is equal to 53.3 maternal deaths per 100,000 LB. In relative terms, the national target is expressed as a 53% reduction in the national MMR between 2014 and 2030. Knowing the departmental distribution of the MMR for 2030 made it possible to calculate the value for each stratum, expressed as an average weighted by the number of live births in each stratum (projected for 2030). Table 3 shows the abbreviated distribution of the MMR in the baseline and target years in Guatemala’s four strata, by stratification criteria and the proportional progressivity factor assigned to the AAPC for each stratum.

**TABLE 2.** Maternal mortality ratio (MMR, per 100,000 live births) values in 2014 and projected targets for 2030 in Guatemala, by departments

**Stratum**	**Departments**	**MMR in 2014**	**MMR projected for 2030**
Stratum 1	Huehuetenango	232.6	88.7
	Totonicapán	167.7	63.9
	Quiche	162.0	61.8
	Petén	149.7	57.1
Stratum 2	Sacatepequez	138.5	62.0
	Izabal	131.8	59.0
	Chiquimula	130.6	58.5
	Chimaltenango	129.2	57.9
	San Marcos	127.8	57.2
	Alta Verapaz	123.9	>55.5
	Jalapa	114.0	51.0
Stratum 3	Sololá	97.9	60.5
	Baja Verapaz	97.9	60.5
	Quetzaltenango	85.0	52.5
	Jutiapa	74.3	45.9
	Santa Rosa	71.9	44.4
	Escuintla	65.3	40.3
	Suchitepequez	62.1	38.3
	Retalhuleu	59.5	36.7
Stratum 4	Guatemala	48.0	34.8
	Zacapa	31.6	22.9
	El Progreso	23.4	17.0

***Source:*** Prepared by the authors.

**TABLE 3.** Average annual percent change in MMR by stratum, calculated from the department-specific maternal mortality ratio (MMR)^a^ recorded in 2014 and 2030 projected value in Guatemala

Stratum^b^	MMR		Average annual percentage change
	2014	2030	
Stratum 1 (departments with MMR ≥ 140)	200.2	76.4	-6.4
Stratum 2 (departments with MMR between 114.1 and 140)	12.0	56.9	-5.4
Stratum 3 (departments with MMR between 57 and 114)	76.1	47.0	-3.2
Stratum 4 (departments with MMR < 57)	45.4	32.9	-2.1

***Source:*** Prepared by the authors.

a MMR is expressed per 100,000 live births.

b The departments in each stratum are shown in Table 2.

**TABLE 4.** Baseline values and average and distributional maternal mortality ratio (MMR)^a^ targets for 2030 in Guatemala

MMR summary measure	2014	2030
National average	113.0	53.3
Absolute inequality gap	154.8	43.4
Relative inequality gap	4.4	2.3

***Source:*** Prepared by the authors.

a MMR is expressed per 100,000 live births.

Finally, using the MMR values for the baseline and target years for strata 1 and 4 (i.e., the bottom and top strata of the abbreviated distribution: 200.2 and 45.4 maternal deaths per 100,000 LB in the baseline year, respectively, and 76.4 and 32.9 maternal deaths per 100,000 LB in 2030, respectively), the inequality gaps (AG and RG) for these two points in time were calculated, as shown in Table 4. In addition to the national average reduction target, distributional targets for the country for reducing inequality in MMR were calculated by applying equations [4] and [5], which yielded values of 43.4 and 2.3 for the AG and RG, respectively, in 2030 (Table 4). If the MMR continues to decline at the same pace as between 2009 and 2014 and if the country’s actions are focalized with territorial progressivity, Guatemala could reduce its national MMR by 53%, its absolute geographic inequality gap by 72%, and its relative geographic inequality gap by 48% by 2030.

## DISCUSSION

The methodology described in this article has been shown to be an intuitive, robust, and practical means of making the commitment to “leave no one behind” explicit in national and regional efforts aimed at achieving the 2030 Agenda goals and, specifically, SDG3: “ensure healthy lives and promote well-being for all at all ages”.

**FIGURA 1.**
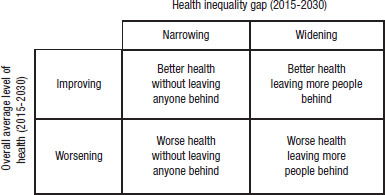
Schematic framework for monitoring progress towards the achievement of Sustainable Development Goal 3 with equity

***Source:*** Prepared by the authors, based on Minujin and Delamónica (17) and Mújica (18).

In addition to the customary target of improving the overall average value of the indicators, setting measurable targets for reducing inequality is the first step towards strengthening accountability and, through the monitoring of these targets, informing decisions, policies, and interventions aimed at achieving SDG3 with equity. In fact, the methodology presented here adapts into the current context an approach originally proposed by the United Nations Children’s Fund (UNICEF) in 2002, and operationalizes it in a monitoring framework, as shown in Figure 1 (17, 18). Under this framework, in a given population setting, it is essential to simultaneously monitor changes in both health level and distribution: only when the improvement in the territorial average is coupled with a reduction in its distributive inequality will it be possible to achieve the desired scenario of improving population health leaving no one behind.

In general, while the formulation of goals aimed at achieving greater health equity is a global phenomenon, the practice of formulating explicit targets for reducing health inequalities has not yet become a standard part of health planning processes in all regions, as it has in Western European countries, especially the United Kingdom (19). In the Americas, the most visible efforts in this regard have been catalyzed by the Pan American Health Organization (20–22), most notably in its last two six-year strategic plans (23, 24), in which Member States adopted explicit targets for the reduction of social inequalities in health, in line with the first objective of the Organization’s mission: to promote equity in health. The advent of the 2030 Agenda, with its SDGs––coupled with the lessons learned from the implementation of the UN Millennium Declaration, with its MDGs (25) ––has renewed the sense of urgency regarding the need to put in place systems to monitor social inequalities, including health inequalities (26, 27). The recent final report of the Commission on Equity and Health Inequalities in the Americas, chaired by Sir Michael Marmot, highlights in its recommendation 11A the need to “make health equity a key indicator of societal development and establish mechanisms of accountability,” and recognizes monitoring of health inequalities as a priority governance mechanism for achieving health equity (28).

The TSA_SDG3 is intended to serve only as a practical and flexible guide for promoting specific institutional actions based on the quantification of desired and feasible changes in the distribution of health and well-being and to inform decision-making aimed at meeting SDG3. Flexibility is built into at least three central aspects of this algorithm: (a) the level of data disaggregation, as the algorithm can serve both the local level (such as the neighborhoods of a city) and the regional level (the countries of a continent or region); (b) the criterion for stratifying territorial units, i.e., the selection of cut-points in the baseline distribution of the health indicator, for which there are alternatives to the method presented here; and (c) the proportional progressivity criterion applied to the projected rate of change—i.e., the AAPC—in the health indicator by 2030, for which there are also multiple methodological options and which essentially reflect the degree of aversion to inequality exhibited by each society (29). These three central aspects, among others, can be seen as direct opportunities for engaging in interdisciplinary and intersectoral dialogue and reaching consensus at the relevant levels necessary to implement this proposal. Setting targets to reduce health inequalities should not be seen as a mathematical–statistical exercise, but rather as a collaborative effort in the collective pursuit of equity. In the practical example given here, the application of the TSA_SDG3 could lead Guatemala to implement dialogue- and consensus-based policies that would not only halve the MMR by 2030 (in this case reducing it from 113 per 100,000 LB to 53 per 100,000 LB), but would also, at the same time, drastically reduce subnational geographic inequality in the risk of maternal death, narrowing the absolute inequality gap by three quarters (i.e., from 155 to 44 per 100,000 LB) and halving the relative inequality gap (i.e., from 4.4 to 2.3), with the consequent health and social benefits that this would entail.

Effective application of the algorithm is dependent on the quality of the available data. This could be a limitation, especially for the use of subnationally disaggregated administrative data, if standard critical and quality control procedures (30) are not first applied to the crude data (potential problems include underreporting in vital statistics, misclassification of deaths, and instability of small numbers). As in all analytical work, it is recommended that the data used to formulate health inequality reduction targets reflect the principles of study base, deconfounding, and comparable accuracy (31). Another limitation of the proposed algorithm may arise from the presence of the residual bias inherent in ecological study designs, especially when using data from administrative sources. Using data with the greatest possible geospatial disaggregation is therefore recommended.

Notwithstanding these limitations, the proposed methodology provides a direct territorial correlation with regard to the behavior of SDG3 indicators, while also making it possible to identify the territorial units that have the greatest health inequalities and are therefore at highest risk of “falling behind.” This provides valuable information for setting territorial targets, making decisions, and implementing appropriately targeted strategies and interventions.

The sense of urgency regarding the need to address and eliminate unfair inequalities in opportunities for health and well-being as part of the effort to achieve universal health and sustainable development––as globally agreed in the 2030 Agenda––has been suddenly and dramatically heightened by the emergence of the new SARS-CoV-2 coronavirus and the COVID-19 pandemic, which have exposed and amplified social and, especially, health inequalities (32). The path to meeting the 2030 targets now has post-pandemic characteristics and, consequently, society as a whole will need to review and rethink its priorities. In this process, it is necessary to affirm the primacy of the principle of health equity for the achievement of SDG3 and to renew the commitment not to leave anyone behind. Decisions and actions aimed at achieving SDG3, especially in the post-pandemic scenario, will be better informed if, aside from national average progress, they are guided by explicit SDG3-related health inequality reduction targets.

## Disclaimer.

Authors hold sole responsibility for the views expressed in the manuscript, which may not necessarily reflect the opinion or policy of the RPSP/PAJPH and/or PAHO.
